# A case of cardiac strangulation following epicardial pacemaker implantation

**DOI:** 10.1007/s11748-020-01337-y

**Published:** 2020-04-08

**Authors:** Chihiro Miyagi, Yoshie Ochiai, Yusuke Ando, Manabu Hisahara, Hironori Baba, Tomoya Takigawa, Shigehiko Tokunaga

**Affiliations:** Department of Cardiovascular Surgery, Japan Community Health Care Organization (JCHO), Kyushu Hospital, 1-8-1, Kishinoura, Yahatanishi-ku, Kitakyushu, 806-8501 Japan

**Keywords:** Cardiac strangulation, Epicardial pacemaker, Pediatric

## Abstract

An 8-year-old boy had undergone permanent epicardial pacemaker implantation with a Y-shaped bipolar ventricular lead on day 6 after birth for treatment of congenital complete atrioventricular block. He was found to have pulmonary stenosis and mitral stenosis by follow-up echocardiography. Further studies including computed tomography and cardiac catheterization revealed that the pacemaker lead had completely encircled the cardiac silhouette and was in a state of “cardiac strangulation”. We removed the previous pacing leads and generator and implanted a new epicardial dual-chamber pacing system in the right atrium and right ventricle. Additionally, an expanded polytetrafluoroethylene sheet was placed between the new leads and the heart to prevent recurrence of cardiac strangulation.

## Introduction

Cardiac strangulation is a mechanical complication that occurs when epicardial pacemaker leads tighten around the heart. As a patient grows, cardiac strangulation can lead to coronary stenosis, ventricular dysfunction, or right ventricular outflow tract obstruction with a risk of cardiac arrest and sudden death [1–4]. We herein describe an 8-year-old boy who had undergone implantation of an epicardial ventricular pacing lead as a neonate for congenital complete atrioventricular block and later presented with suspected cardiac strangulation on echocardiography. He underwent successful pacemaker revision.

## Case

**Fig. 1:**
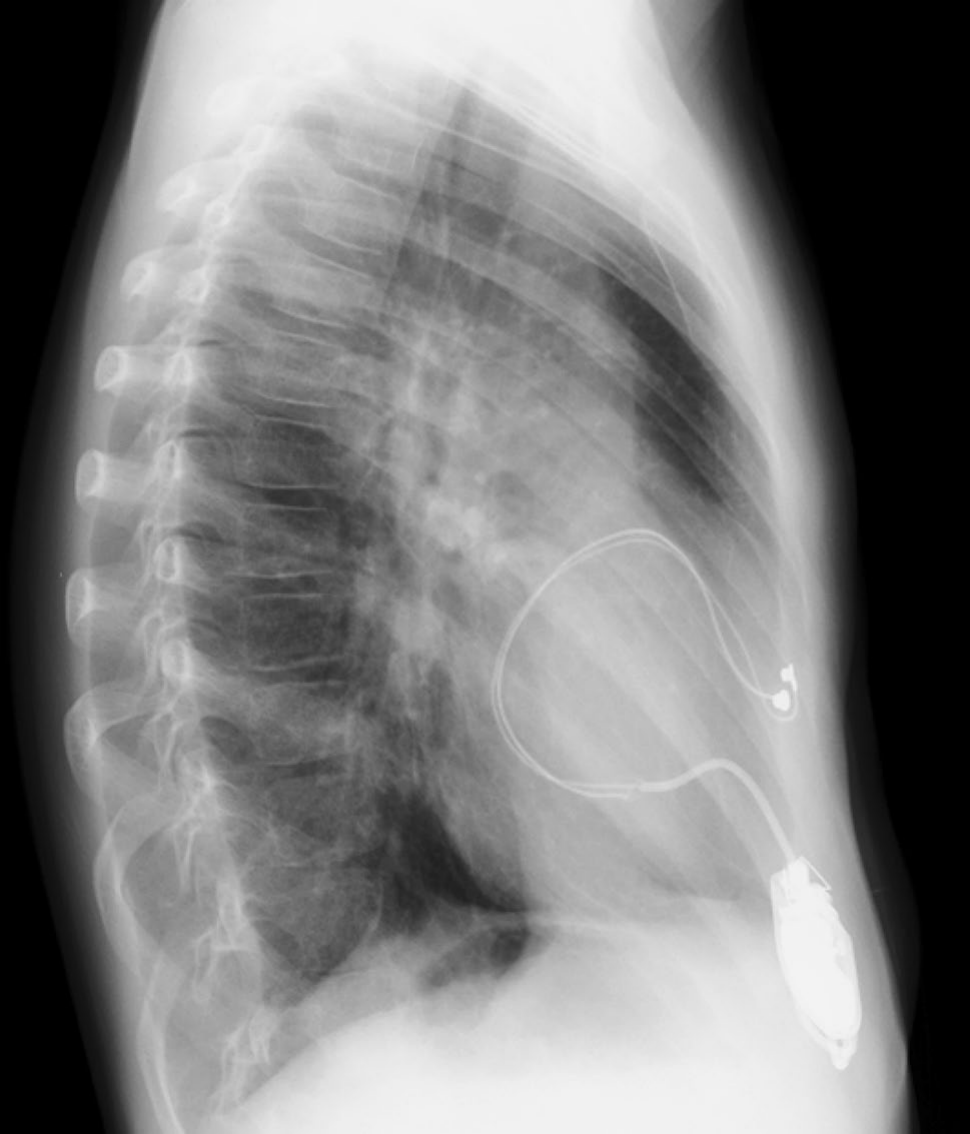
Lateral view of the X-ray at 8 years of age when we noticed the cardiac strangulation

**Fig. 2:**
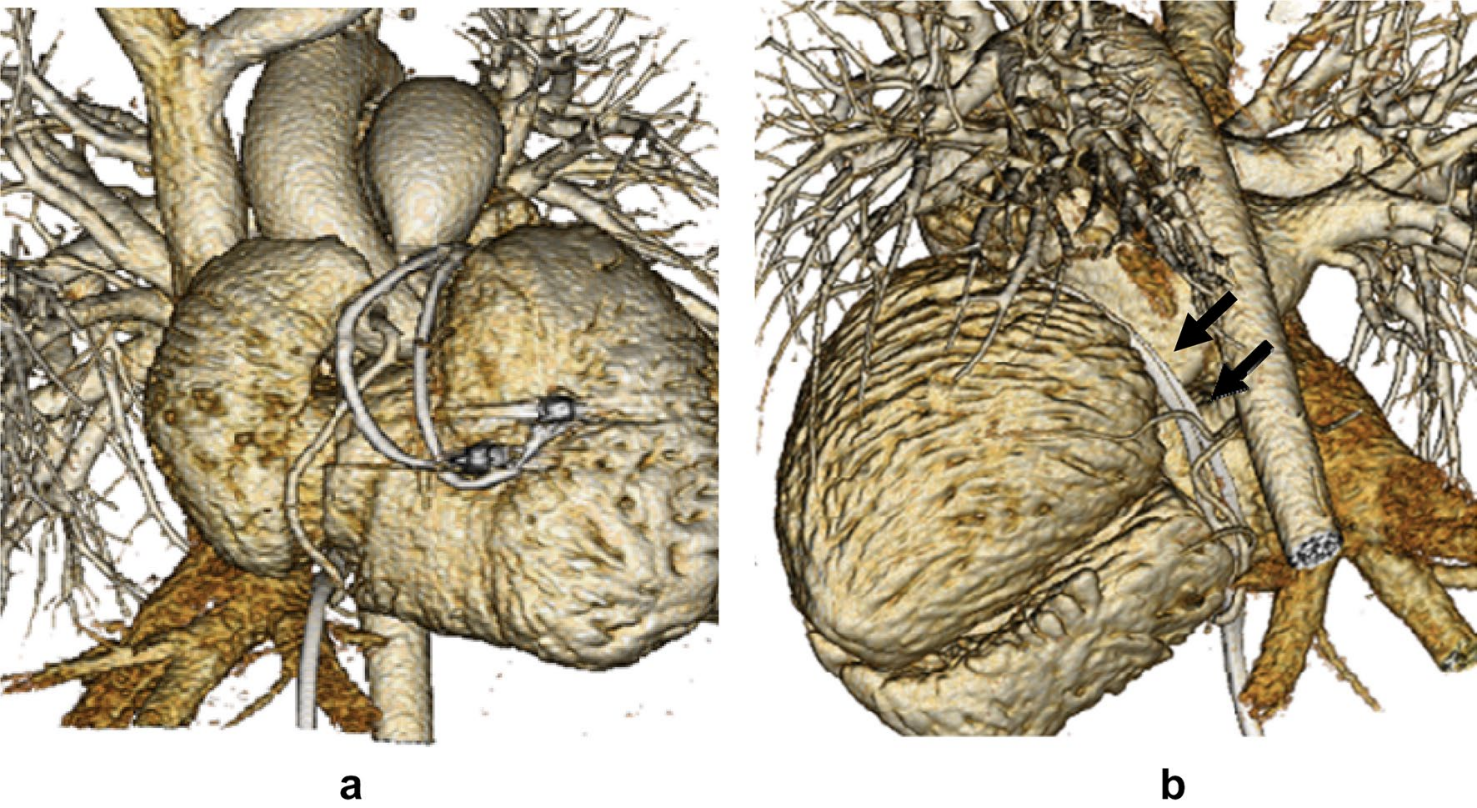
Three-dimensional computed tomography images. **a** The frontal view shows the epicardial leads compressing the main pulmonary artery. **b** The lateral view shows the leads extending down along the atrioventricular groove (arrows)

**Fig. 3:**
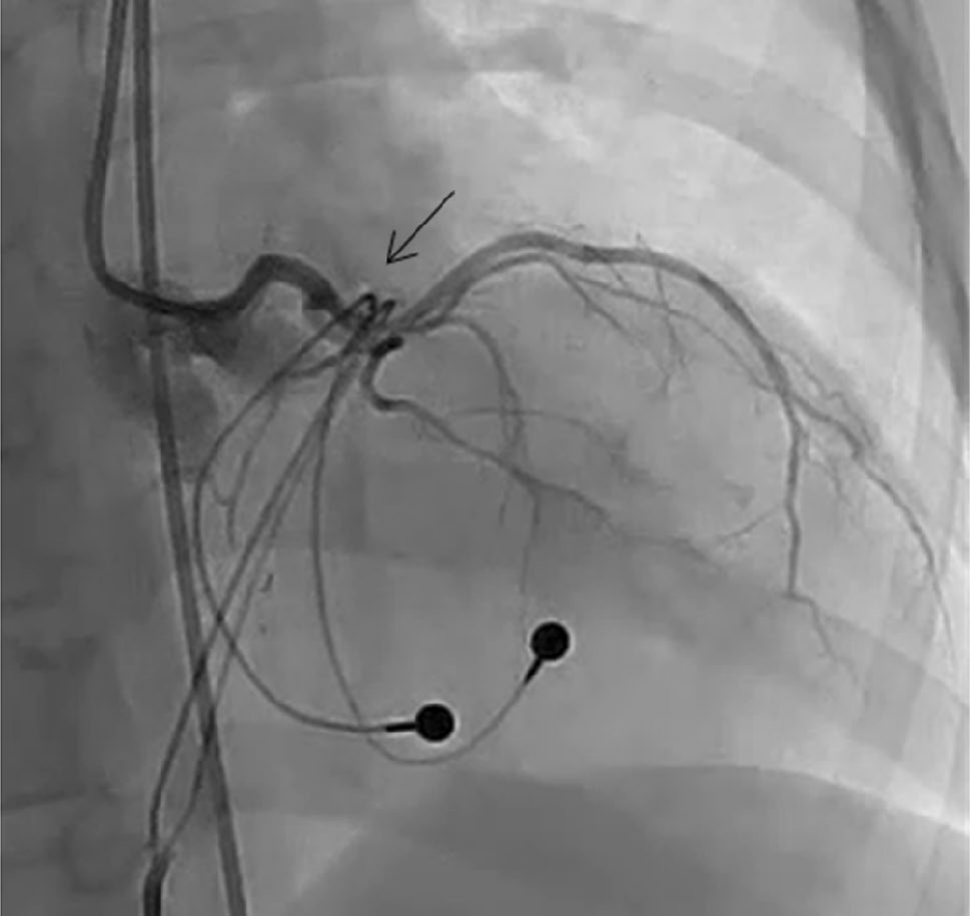
Angiography demonstrating compression of the left anterior descending coronary artery by the epicardial pacing leads (arrow)

The patient was an 8-year-old boy with an antenatal diagnosis of congenital complete atrioventricular block secondary to maternal anti-Sjögren’s syndrome-related antigen A antibody. He was born at term by caesarean section and weighed 2744 g with a ventricular rate of 50 beats/min. He had low cardiac output due to bradycardia, which was unaffected by inotropes or infusion of diuretics. Therefore, on day 6 after birth, he underwent permanent epicardial pacemaker implantation with a bipolar Y-shaped ventricular lead via a lower median sternotomy. The excess leads were rolled up and positioned in front of the heart in the pericardium without fixation, then connected to a VVI generator in the median rectus sheath. The pericardium was closed. He presented for routine follow-up care in our hospital annually without chest pain or dyspnea. When the patient reached 5 years of age, we performed generator exchange uneventfully. At 8 years of age, he was admitted to our hospital for catheter treatment of tiny patent ductus arteriosus. He had a systolic ejection murmur with no clinical symptoms. His lateral chest radiograph (Fig. 1) revealed that the pacemaker lead was completely wrapped around the cardiac silhouette, and we became concerned about cardiac strangulation. Echocardiography showed that the pacemaker leads were located at the level of the supra-pulmonary valve and atrioventricular groove, causing constriction of the pulmonary and mitral valves. A subsequent computed tomography scan confirmed the presence of cardiac strangulation by the epicardial pacing leads (Fig. 2a, b). Cardiac catheterization with coronary angiography was performed to better delineate the pathway of the pacing leads. The systolic pressure gradient at the main pulmonary artery (MPA) and mitral valve was 33 and 12 mmHg, respectively. The left anterior descending coronary artery was compressed by the leads (Fig. 3).

The patient was scheduled for urgent pacemaker lead revision. A full median sternotomy was performed, and the bipolar Y-shaped ventricular leads were found to be tightly wrapped around the heart, compressing the MPA from the left side. The leads went down behind the atrioventricular groove, and both leads appeared to the front from the left side of the inferior vena cava. After peeling off the adhesions around the epicardial leads, we dissected and completely removed them without using the cardiopulmonary bypass. The compression of the MPA was released immediately after the lead removal. New bipolar Y-shaped atrial and ventricular leads were then implanted. The excess length of the new leads was positioned in front of the pericardium, and an expanded polytetrafluoroethylene sheet was placed behind the leads to prevent recurrent cardiac strangulation by the leads. The new generator was placed in the previous rectus sheath pocket. He had an uneventful recovery and is asymptomatic on follow-up at 2 years. Contrast computed tomography and echocardiography showed expansion of the MPA and disappearance of the pulmonary and mitral stenosis.

## Discussion

**Fig. 4:**
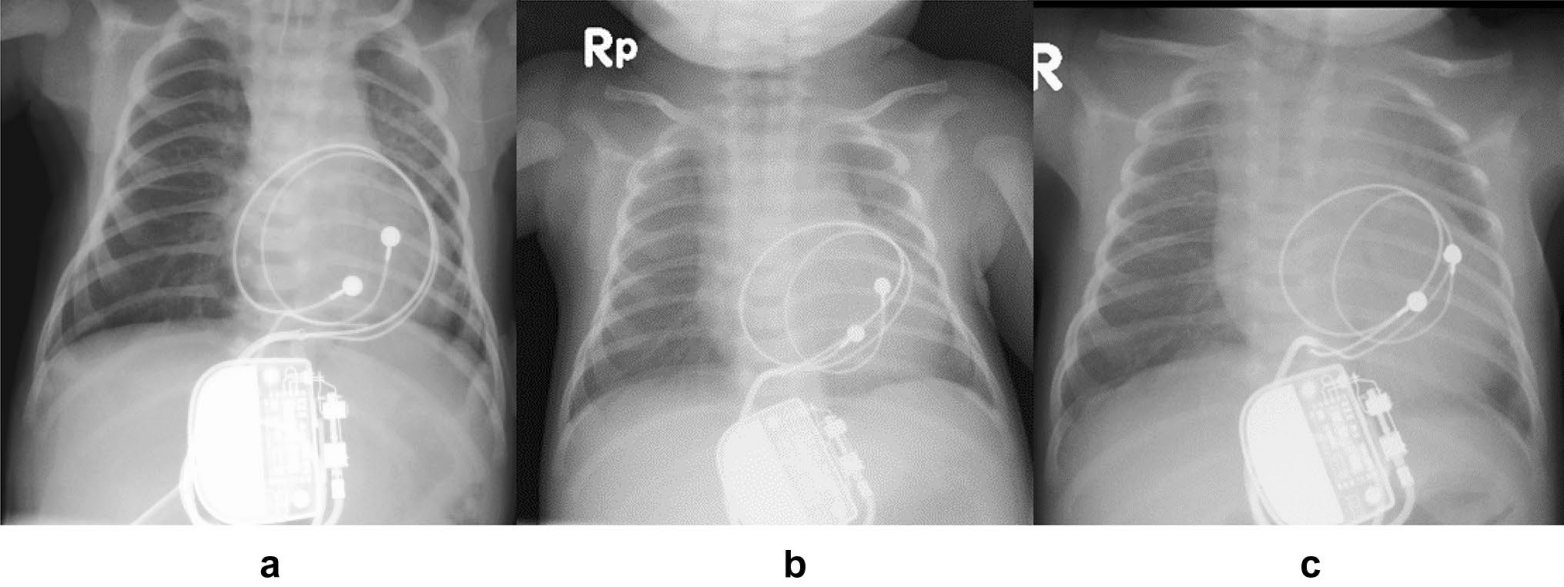
Serial of chest radiographs **a** just after the first implantation of the generator at 6 days of age **b** at postoperative 14th day which might indicate that the leads have already slid beside the main pulmonary artery, and **c** at postoperative 16th day

**Fig. 5:**
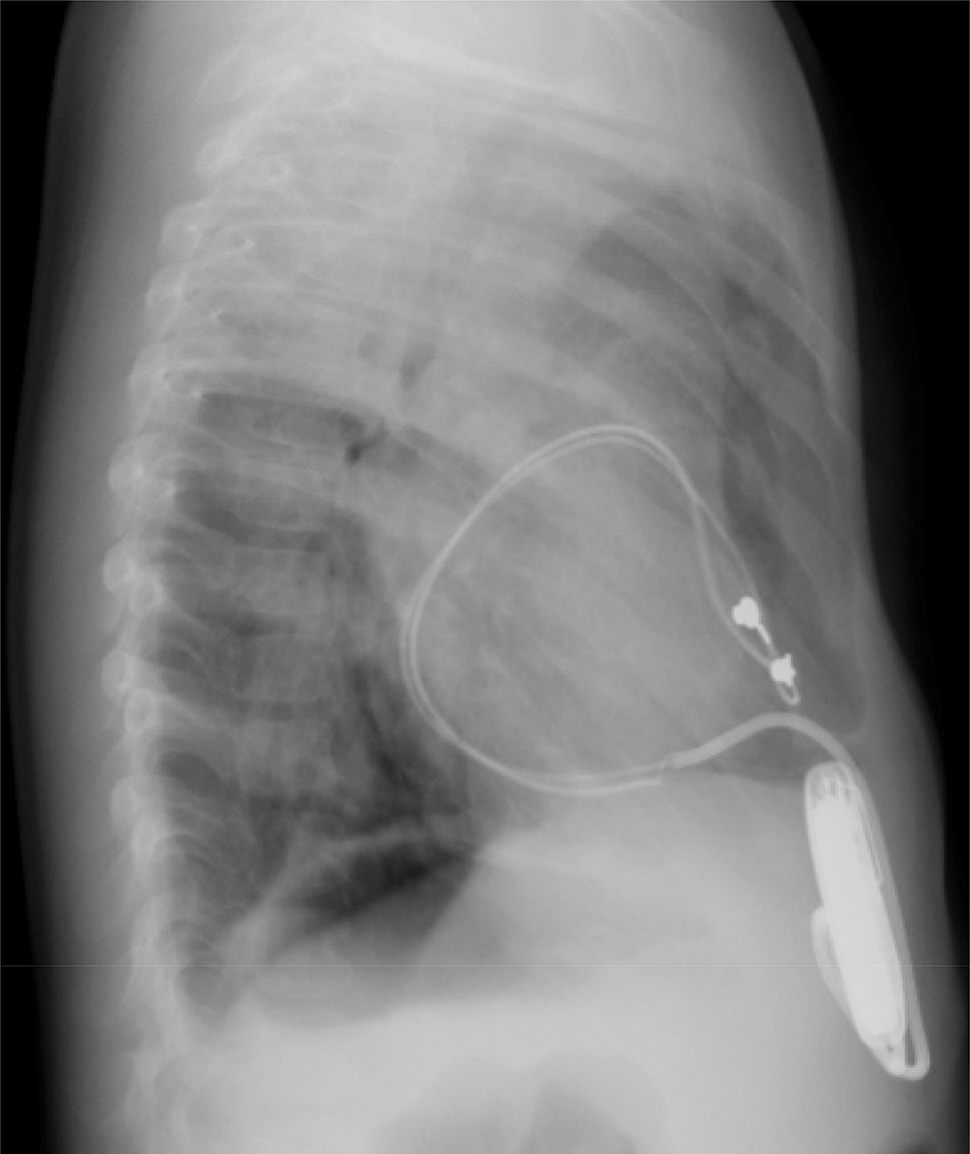
Lateral view of the X-ray which was taken at 11 months of age to diagnose pneumoniae in the other hospital

Cardiac strangulation is a rare and life-threatening complication of epicardial pacemaker leads, and only a few cases have been reported [1–8] (Table 1). Carreras et al. [5] showed that cardiac strangulation lacks consistent diagnostic imaging findings. They also reported that the risk of strangulation is higher in patients aged ≤ 6 months. When we reviewed the past data, the frontal view of chest X-ray just after the first implantation (Fig. 4a) and serial X-ray images (Fig. 4b, c) implied that the leads had already slid backward of the heart on 14th postoperative day. At initial implant, his weight was only 2.5 kg. Probably, the large loop of the leads slipped from the left side of the MPA to behind his small heart soon after the initial implant. Unfortunately, we did not check the lateral side of the X-ray immediately after the first implant. In the other hospital at his 11 months of age, the lateral view of the chest X-ray which had been taken due to pneumoniae proved the leads had moved backwards to the heart (Fig. 5). In the present case, opportunity of catheter treatment of tiny patent ductus arteriosus led us to the diagnosis of cardiac strangulation, and any fatal complication such as myocardial ischemia did not occur until surgical revision of the epicardial leads at 8-year-old fortunately. However, we should have been suspicious about the cardiac strangulation only by the serial frontal X-ray and obtained the lateral X-ray soon after the initial implant.

**Table 1:** Case series of cardiac strangulation

Case	Year	Location	Age at PMI	Age at CS	Primary diagnosis	Compression	Symptom	Outcome
1	1988	USA [2]	6 days	20 months	CAVB	RCA, PA	syncope	Alive
2	1992	Japan [1, 5]	3 years	9 years	TOF	LCA, PA	CHF	Unknown
3	1997	Belgium [3, 5]	8 months	6 years	VSD	Apex	Chest pain	Death
4	2000	Japan [5]	2 days	10 months	CAVB	LCA	CHF	Death
5	2000	USA [5]	2 months	5 years	cJET	PA	SM	Alive
6	2007	USA [4]	< 1 month	9 years	Bradycardia	LCA	Chest pain	Alive
7	2007	USA [4]	7 days	12 years	CTGA	LCA, LV	None	Alive
8	2008	Germany [5, 8]	3 months	2 years	CAVB	LCA	CHF	Alive
9	2011	Canada [5, 6]	2 days	3 years	CAVB	LCA	Unknown	Unknown
10	2017	Japan	6 days	8 years	CAVB	PA, AV groove	CHF	Alive

*PMI* pacemaker implantation, *CS* cardiac strangulation, *CAVB* complete atrioventricular block, *RCA* right coronary artery, *PA* pulmonary artery, *TOF* tetralogy of Fallot, *LCA* left coronary artery, *CHF* chronic heart failure, *VSD* ventricular septal defect, *cJET* congenital junctional ectopic tachycardia, *SM* systolic murmur, *CTGA* corrected transposition of great arteries, *LV* left ventricle, *AV* atrioventricular

To prevent cardiac strangulation in neonates or even infants undergoing implantation of an epicardial pacemaker, redundant leads must not be looped very long anteriorly around the cardiac chambers nor placed inside the pericardium. Alhuzaimi et al. [6] reported that excess leads can be placed in the pleural space with the generator implanted on the diaphragmatic surface of the same pleural space. Using an expanded polytetrafluoroethylene sheet to separate the heart and leads or circling tiny counterclockwise loop of lead might be effective method of preventing cardiac strangulation.

## Conclusion

We reported the 8-year-old boy who received the VVI implant on day 6 after birth and developed cardiac strangulation from the leads underwent urgent successful surgical revision of the previous epicardial leads. Careful review of the anteroposterior and lateral chest X-ray image at routine interval is indispensable considering the possibility of cardiac strangulation.

## References

[1] Sugita T, Yokota Y, Ando F (1992). Cardiac strangulation with permanent epicardial pacemaker lead. Kyobu Geka.

[2] Brenner JI, Gaines S, Cordier J (1988). Cardiac strangulation: two-dimensional echo recognition of a rare complication of epicardial pacemaker therapy. Am J Cardiol.

[3] Eyeskens B, Mertens L, Moerman P, Ector H, Daenen W, Gewilling M (1997). Cardiac strangulation, a rare complication of epicardial pacemaker leads during growth. Heart.

[4] Salerno JC, Johnston TA, Chun TU, Jones TK (2007). Coronary compression by an epicardial pacing lead within the pericardium. J Cardiovasc Electrophysiol.

[5] Carreras EM, Duncan WJ, Djurdjev O, Campbell AI (2015). Cardiac strangulation following epicardial pacemaker implantation: a rare pediatric complication. J Thorac Cardiovasc Surg.

[6] Alhuzaimi A, Roy N, Duncan WJ (2011). Cardiac strangulation from epicardial pacemaker: early recognition and prevention. Cardiol Young.

[7] Macicek SL, Cannon BC, Kyle WB, Krishnamurthy R, Breinholt JP, Ing FF (2011). Dynamic coronary artery compression by pacemaker lead. Circulation.

[8] Riede FT, Kostelka M, Dahnert I (2009). Cardiac strangulation: a rare, but devastating complication of epicardial pacing causing progressive myocardial ischemia. Eur Heart.

